# Compensation for the Public Services of Medical Practitioners

**Published:** 1850-04

**Authors:** 


*Compensation of the public services of Medical Practitioners.*—A newspaper has been forwarded to us containing some excellent remarks on this subject, taken from an annual address to the Cortland County Medical Society, by Dr. F. Hyde. Dr. Hyde states that the sum paid for the medical and surgical attendance upon the poor-house patients of Cortland County for several years past, has been from $15 to $20 annually! Here, however, we presume, as in other instances, the fault lies with the medical profession. Union and co-operation for common interests would obviate this, as well as numerous other evils. This subject was discussed at the last meeting of the New York State Society, and some resolutions adopted, which we shall publish when the printed transactions of the Society are received.

